# Interactive Effect of Age on Overall and Relative Survival Benefits of Radiotherapy for Early-Stage Diffuse Large B-Cell Lymphoma in the Rituximab Era

**DOI:** 10.14740/jh2134

**Published:** 2026-02-20

**Authors:** Xin Wang, Xin Liu, Qiu Zi Zhong, Yun Peng Wu, Tao Wu, Yong Wen Song, Bo Chen, Hao Jing, Yuan Tang, Jing Jin, Yue Ping Liu, Hui Fang, Ning Ning Lu, Ning Li, Yi Rui Zhai, Wen Wen Zhang, Yong Yang, Shu Lian Wang, Shu Nan Qi, Ye Xiong Li

**Affiliations:** a Department of Radiation Oncology, National Cancer Center/National Clinical Research Center for Cancer/Cancer Hospital, Chinese Academy of Medical Sciences (CAMS) and Peking Union Medical College (PUMC), Collaborative Innovation Center for Cancer Medicine, Beijing 100021, China; b Department of Radiation Oncology, Shandong Cancer Hospital and Institute, Shandong First Medical University and Shandong Academy of Medical Sciences, Jinan 250117, Shandong, China; c Beijing Hospital, National Geriatric Medical Center, Beijing, China; d Affiliated Hospital of Guizhou Medical University, Guizhou Cancer Hospital, Guiyang, Guizhou, China; e Department of Radiation Oncology, National Cancer Center/National Clinical Research Center for Cancer/Cancer Hospital and Shenzhen Hospital, Chinese Academy of Medical Sciences (CAMS) and Peking Union Medical College (PUMC), Shenzhen 518116, China; f Department of Radiation Oncology, Fujian Medical University Union Hospital, Fuzhou, Fujian, China; g These authors contributed equally to the work.

**Keywords:** DLBCL, Radiotherapy, Relative survival, Age

## Abstract

**Background:**

The aim of the study was to determine the interactive effect of age on overall survival (OS) and relative survival (RS) benefits of radiotherapy (RT) in early-stage diffuse large B-cell lymphoma (DLBCL).

**Methods:**

Data for 10,841 adults with early-stage DLBCL from the Surveillance, Epidemiology, and End Results database between 2002 and 2015 were retrospectively analyzed. Primary therapy was classified as combined-modality treatment (CMT; n = 3,631) and chemotherapy alone (n = 7,210). Inverse probability of treatment weighting was used to balance covariate distribution between the treatment groups. Survival was estimated and compared using the Kaplan-Meier method and log-rank test, respectively. Age-RT interactive effect on survival was examined through Cox regression multiplicative interaction analysis.

**Results:**

Using age of 60 years as the reference, older age was an independent predictor of shorter OS in the multivariable Cox model (hazard ratio (HR), 1.07; 95% confidence interval (CI), 1.06–1.07; P < 0.001). After controlling for background mortality, older age was not an independent predictor of RS (HR, 1.00; 95% CI, 0.99–1.00; P = 0.842). Across all age groups, patients treated with CMT had better OS and RS than those who received chemotherapy alone. A significant interaction between age and RT was identified for both OS (P_interaction_ = 0.020) and RS (P_interaction_ = 0.038), indicating greater RT benefit in young patients. A linear correlation existed between RS and OS at the treatment arm level.

**Conclusions:**

RT was associated with improved net survival across all ages, particularly for young adults. RS was a valid alternative endpoint for prognostication and benefit evaluation.

## Introduction

Diffuse large B-cell lymphoma (DLBCL) is the most common subtype of aggressive non-Hodgkin lymphoma (NHL) [1]. At initial diagnosis, 25–30% of the patients are in early stages, having an excellent prognosis with 10-year overall survival (OS) rates of 70–90% following curative treatment [2]. However, OS consistently decreases with longer follow-up durations after initial treatment because of non-lymphoma-related deaths (non-LRDs), including background or treatment-related deaths [3]. Rituximab was approved by the US Food and Drug Administration (FDA) in the early 2000s for the frontline treatment of DLBCL. Previous studies generally regard the period following its approval and clinical application as the rituximab era. Rituximab combined with CHOP (cyclophosphamide, doxorubicin, vincristine, and prednisone) or CHOP-like chemotherapy regimens, with or without consolidative radiotherapy (RT), is considered the standard therapy for early-stage DLBCL [4–7].

The role of consolidative RT in the management of early-stage DLBCL remains controversial [8]. A few randomized studies have successfully omitted RT to minimize long-term toxicity while maintaining excellent outcomes [9–11]. However, the patients in these studies were highly selective and did not reflect the real-world DLBCL patient population, such as elderly patients and those with comorbidities. Real-world data reported an OS benefit of combined-modality treatment (CMT) ranging from 4% to 13% compared to chemotherapy alone [3, 12–14]. In a recent comprehensive meta-analysis, we demonstrated that the OS benefit of RT was related to progression-free survival (PFS) after chemotherapy for DLBCL [15]. The OS benefit of RT was more pronounced in patients at intermediate or high risk of progression (PFS ≤ 80%) but was limited in those at low risk (PFS > 80%).

DLBCL commonly occurs in elderly patients, with a median age at diagnosis exceeding 60 years [1]. These individuals have been reported to experience significantly worse outcomes [16]. Prognostic models for DLBCL invariably include old age as an independent risk factor [17–21], but most of these large cohort studies have used crude survival indices, such as OS, as an endpoint, while failing to fully elucidate how treatment affects outcomes in DLBCL patients due to potential bias from non-LRDs. Determining the cause of death can be challenging over a long-term follow-up, particularly among elderly individuals having multiple comorbidities. Relative survival (RS) circumvents the confounding factors by using the expected mortality hazard from the general population as a reference to accurately reflect the DLBCL-related survival loss and the relationship between age and treatment benefit [3, 22, 23]. In a recent study [3], we demonstrated that RT was associated with reduced LRDs and improved long-term RS in early-stage DLBCL. In this predominantly elderly population, it is critical to explore how age affects prognosis and the net survival benefit of treatment.

This study analyzed the Surveillance, Epidemiology, and End Results (SEER) database to investigate the age-dependent, interactive effect of RT on OS and RS benefits for early-stage DLBCL in the rituximab era.

## Materials and Methods

### Eligibility criteria and study population

We performed a retrospective analysis of data from early-stage DLBCL patients registered in the SEER database in the rituximab era between 2002 and 2015. As detailed previously [3], early-stage patients who underwent CMT (chemotherapy and RT) or chemotherapy alone were eligible for inclusion.

Age was analyzed as a continuous variable and was also categorized into four age groups: 18–40, 41–60, 61–80, and > 80 years. The year of diagnosis was categorized as 2002–2008 or 2009–2015. The cause of death was classified as LRD or non-LRD based on the variables “SEER cause-specific death classification” and “SEER other cause of death classification,” obtained from the SEER database.

### Endpoints

The primary endpoints were RS and OS. RS was defined as net survival in the absence of other causes of death (non-LRDs), and was calculated as the ratio of actual survival to the expected survival for equivalent groups from the age-, sex-, and calendar year-matched general US population using the Ederer II method [24]. OS was measured from the date of diagnosis to the date of death from any cause. Based on the need for a 10-year follow-up to identify the maximal survival difference and treatment benefit [3], 10-year OS and RS values were considered the primary benchmark to assess increased risk of mortality. LRD was defined as DLBCL-related deaths not resulting from other causes or long-term treatment toxicity. Standardized mortality ratio (SMR) was defined as the ratio of actual mortality in the study cohort to the expected mortality in the matched general US population. A SMR > 1.0 indicated a worse than expected survival.

### Statistical analysis

The cumulative mortality incidence according to the cause of death was calculated through competing risk analysis using Gray’s test. Survival was analyzed using the Kaplan-Meier method and compared between treatment groups using the log-rank test. Differences among categorical variables were assessed using the Chi-square test. Penalized splines were fitted in the multivariate Cox model to smooth out the instantaneous probability (hazard ratio (HR)) of survival according to age as a continuous variable. Inverse probability of treatment weighting (IPTW) was applied using propensity scores to balance the treatment arms [25]. Standardized mean differences were used to assess the balance of covariates, with a value of < 0.1 considered acceptable [26]. After adjusting for covariates using IPTW, a transformed Cox regression model for RS was used to examine excess mortality related to the treatment arms [27]. The correlation between OS and RS across subgroups was estimated using Pearson’s correlation coefficient (r) in linear regression. Age-RT interaction on survival was examined through Cox regression multiplicative interaction analysis [28]. Statistical analyses were performed using the compareGroups, cmprsk, cmprskcoxmsm, ipw, survival, relsurv, survminer, popEPi, simPH, and smoothHR packages in R, version 4.3.1. Data from the SEER database (SEER Research Plus Data, 17 Registries, Nov 2021 Sub (2000–2019)) were queried using SEER*-Stat software, version 8.4.0.

### Ethics approval and Institutional Review Board statement

This study analyzed data obtained from publicly available, fully anonymized datasets; therefore, ethical approval and informed consent were not required in accordance with local guidelines, and Institutional Review Board approval was not applicable.

## Results

### Baseline characteristics and treatments

Table 1 summarizes the clinical characteristics of 10,841 patients stratified into four age groups. The median age at diagnosis was 63 years, with a range of 18–100 years. The male-to-female ratio was 1.17:1. A small proportion of patients were aged ≤ 40 years (n = 1,401; 12.9%) or > 80 years (n = 1,363; 12.6%). Stage-I disease was detected in 47.8% of the patients.

**Table 1:** Baseline Characteristics of Early-Stage DLBCL Patients, Stratified by Age Groups at Diagnosis (2002–2015)

Characteristic	18–40 years, n (%)	41–60 years, n (%)	61–80 years, n (%)	> 80 years, n (%)	P
Total	1,401 (100)	3,338 (100)	4,739 (100)	1,363 (100)	
Sex					< 0.001
Male	756 (54.0)	2,009 (60.2)	2,454 (51.8)	622 (45.6)	
Female	645 (46.0)	1,329 (39.8)	2,285 (48.2)	741 (54.4)	
Race					< 0.001
White	1,044 (74.5)	2,659 (79.7)	3,988 (84.2)	1,203 (88.3)	
Black	182 (13.0)	335 (10.0)	255 (5.4)	35 (2.6)	
Other/unknown	175 (12.5)	344 (10.3)	496 (10.5)	125 (9.2)	
Stage					0.001
I	649 (46.3)	1,515 (45.4)	2,335 (49.3)	681 (50.0)	
II	752 (53.7)	1,823 (54.6)	2,404 (50.7)	682 (50.0)	
B symptoms					< 0.001
Yes	366 (26.1)	695 (20.8)	862 (18.2)	246 (18.0)	
No	766 (54.7)	1,865 (55.9)	2,709 (57.2)	765 (56.1)	
Unknown	269 (19.2)	778 (23.3)	1,168 (24.6)	352 (25.8)	
Primary site					< 0.001
Nodal	969 (69.2)	2,101 (62.9)	2,783 (58.7)	766 (56.2)	
Extranodal	432 (30.8)	1,237 (37.1)	1,956 (41.3)	597 (43.8)	
Marital status					< 0.001
Married	605 (43.2)	2,030 (60.8)	2,943 (62.1)	618 (45.3)	
Divorced/separated/widowed	63 (4.5)	455 (13.6)	1,083 (22.9)	599 (43.9)	
Unmarried/single	674 (48.1)	724 (21.7)	491 (10.4)	87 (6.4)	
Unknown	59 (4.2)	129 (3.9)	222 (4.7)	59 (4.3)	
Period					0.026
2002–2008	668 (47.7)	1,550 (46.4)	2,079 (43.9)	631 (46.3)	
2009–2015	733 (52.3)	1,788 (53.6)	2,660 (56.1)	732 (53.7)	
Therapy					< 0.001
CT alone	837 (59.7)	2,154 (64.5)	3,264 (68.9)	955 (70.1)	
CMT	564 (40.3)	1,184 (35.5)	1,475 (31.1)	408 (29.9)	
Final outcome					< 0.001
Alive	1,222 (87.2)	2,446 (73.3)	2,296 (48.4)	229 (16.8)	
LRD	143 (10.2)	570 (17.1)	1,256 (26.5)	616 (45.2)	
Death from other cause	36 (2.6)	322 (9.6)	1,187 (25.0)	518 (38.0)	

DLBCL: diffuse large B-cell lymphoma; CMT: combined modality treatment; CT: chemotherapy; LRD: lymphoma-related death.

Regarding treatment, 3,631 (33.5%) patients received CMT, while 7,210 (66.5%) received chemotherapy alone. The proportion of patients receiving initial RT declined with age, with 40.2%, 35.5%, 31.1%, and 29.9% in the 18–40, 41–60, 61–80, and > 80 years age groups, respectively. After adjusting for IPTW, the treatment groups had similar baseline characteristics.

### Influence of age on causes of death and survival

**Figure 1:**
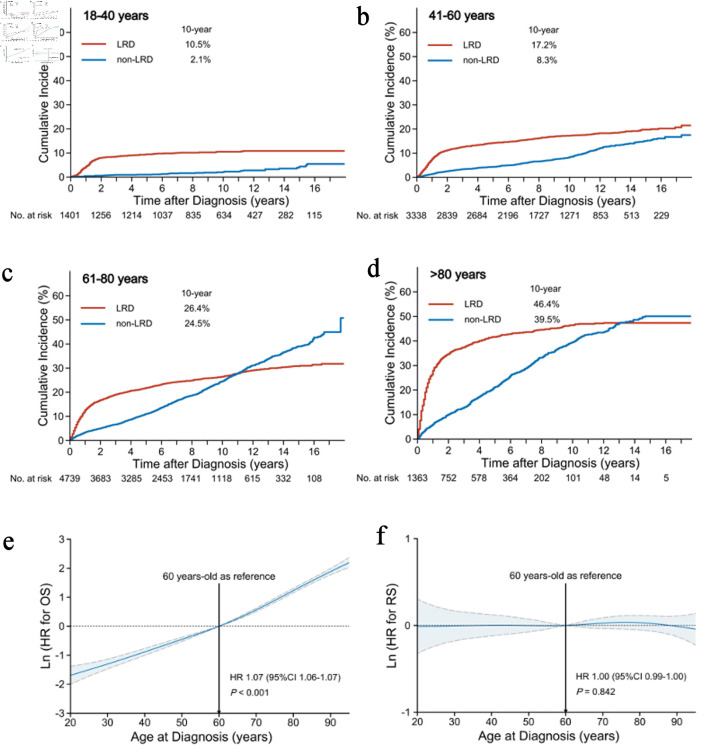
Influence of age on causes of death and survival. Cumulative incidence of LRDs and non-LRDs in the age groups of < 40 years (a), 41–60 years (b), 61–80 years (c), and > 80 years (d). Association of age (as a continuous variable) with OS (e) and RS (f) based on the reference age of 60 years. The mean HRs and 95% CIs are represented by solid and dashed lines, respectively. LRD: lymphoma-related death; non-LRD: non-lymphoma-related death; OS: overall survival; RS: relative survival; CI: confidence interval; HR: hazard ratio.

The LRD and non-LRD profiles varied among age groups. Over a median follow-up of 81 months, 4,648 (42.9%) patients died of DLBCL (2,585/10,841; 23.8%) and other causes (2,063/10,841; 19.0%). The patients generally exhibited a continuously increasing risk for both LRD and non-LRD with age (Fig. 1). Younger patients (≤ 60 years) had a lower risk of LRD and non-LRD than older patients but remained at a consistently higher risk for LRD than for non-LRD during the follow-up (Fig. 1a, b). However, older patients (> 60 years) had a consistently higher risk for non-LRD during the follow-up. Subsequently, beyond 12 years, these patients were at a higher risk of mortality from causes other than DLBCL (Fig. 1c, d). The 10-year cumulative incidences of LRD and non-LRD in the age groups of 18–40, 41–60, 61–80, and > 80 years were 10.5% and 2.1%, 17.2% and 8.3%, 26.4% and 24.5%, and 46.4% and 39.5%, respectively.

To determine the effect of age on survival outcomes, we further explored how age as a continuous variable affects OS and RS. After adjusting for all clinical covariates in multivariable analysis, older age was an independent adverse factor for OS (HR, 1.07; 95% CI, 1.06–1.07; P < 0.001) (Fig. 1e), without an apparent cutoff point (although 60 years is frequently used as the reference age). In contrast, older age was not an independent predictor of RS (HR, 1.00; 95% CI, 0.99–1.00; P = 0.842) (Fig. 1f). This suggests that, after correcting for background mortality, patient age did not affect RS in DLBCL.

### Survival benefit of RT in age-stratified groups

**Figure 2:**
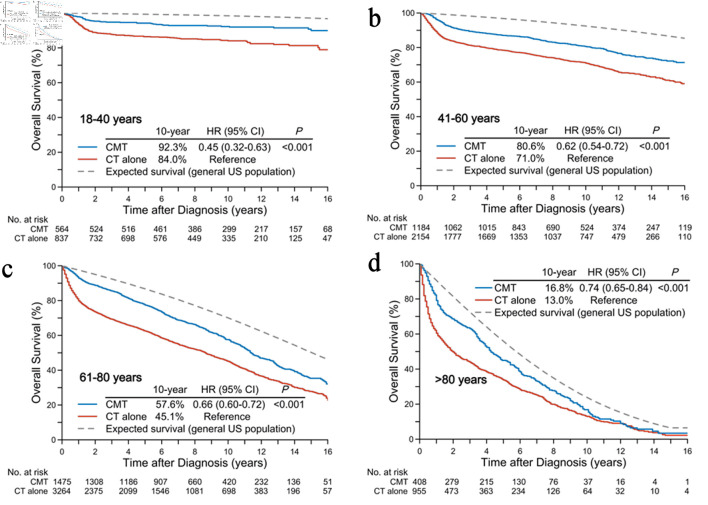
Crude overall and expected survival rates in the general US population according to age groups: < 40 years (a), 41–60 years (b), 61–80 years (c), and > 80 years (d). CMT: combined-modality treatment; CT: chemotherapy.

**Figure 3:**
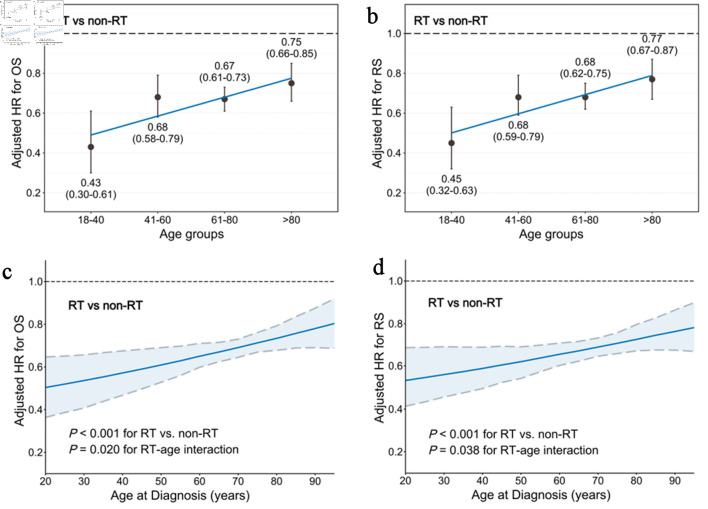
Interaction between age and RT on survival benefits after balancing through IPTW and controlling for clinical covariates. (a) HR for OS (RT vs. non-RT) in the four age groups. (b) HR for RS (RT vs. non-RT) in the four age groups. (c) HR for OS (RT vs. non-RT) according to age as a continuous variable. The simulated plot for the interaction between age and OS efficacy of RT depicts the HR for OS in patients treated with or without RT. (d) HR for RS (RT vs. non-RT) according to age as a continuous variable. The simulated plot for the interaction between age and RS efficacy of RT depicts the HR for RS in patients treated with or without RT. HR: hazard ratio; IPTW: inverse probability of treatment weighting; OS: overall survival; RS: relative survival; RT: radiotherapy.

Figure 2 presents the observed OS in age-stratified patients and the expected OS in the matched general US population. The 10-year observed OS rates for CMT in the age groups of 18–40, 41–60, 61–80, and > 80 years were 92.3%, 80.6%, 57.6%, and 16.8%, compared to 84.0% (HR, 0.45; 95% CI, 0.32–0.63; P < 0.001), 71.0% (HR, 0.62; 95% CI, 0.54–0.72; P < 0.001), 45.1% (HR, 0.66; 95% CI, 0.60–0.72; P < 0.001), and 13.0% (HR, 0.74; 95% CI, 0.65–0.84; P < 0.001) for chemotherapy alone, respectively. After balancing the treatment arms through IPTW, CMT remained associated with improved OS across all age groups (Fig. 3a).

**Figure 4:**
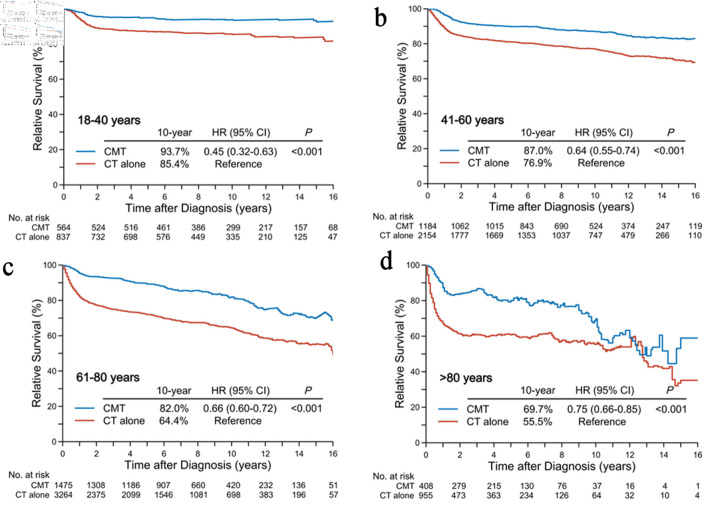
Relative survival differences in patients receiving initial CMT versus chemotherapy alone according to age groups: < 40 years (a), 41–60 years (b), 61–80 years (c), and > 80 years (d). CMT: combined-modality treatment; CT: chemotherapy.

The 10-year RS rates for CMT in the age groups of 18–40, 41–60, 61–80, and > 80 years were 93.7%, 87.0%, 82.0%, and 69.7% (Fig. 4), compared to 85.4% (HR, 0.45; 95% CI, 0.32–0.63; P < 0.001), 76.9% (HR, 0.64; 95% CI, 0.55–0.74; P < 0.001), 64.4% (HR, 0.66; 95% CI, 0.60–0.72; P < 0.001), and 55.5% (HR, 0.75; 95% CI, 0.66–0.85; P < 0.001) for chemotherapy alone, respectively. Balancing with IPTW and adjusting for covariates through Cox multivariable regression analysis also yielded similar results (Fig. 3b).

The SMRs for CMT in the age groups of 18–40, 41–60, 61–80, and > 80 years were 4.43 (95% CI, 3.31–5.94; P < 0.001), 2.80 (95% CI, 2.47–3.17; P < 0.001), 1.62 (95% CI, 1.50–1.75; P < 0.001), and 1.35 (95% CI, 1.21–1.51; P < 0.001), compared to 10.85 (95% CI, 9.17–12.86; P < 0.001), 4.56 (95% CI, 4.22–4.93; P < 0.001), 2.56 (95% CI, 2.44–2.68; P < 0.001), and 1.86 (95% CI, 1.73–1.99; P < 0.001) for chemotherapy alone, respectively. The natural spline analysis revealed a lower SMR for CMT compared to that for chemotherapy alone at all ages up to 90 years (Supplementary Material 1, jh.elmerpub.com).

Subgroup analyses showed robust OS and RS benefits of CMT vs. chemotherapy alone, regardless of age, sex, race, marital status, stage, B symptoms, primary site, and period of diagnosis (Supplementary Material 2, jh.elmerpub.com).

### Interaction between age and RT on survival benefits

We observed that the HRs of RT vs. non-RT for both OS and RS increased with age after balancing the treatment arms through IPTW, but were always less than 1, clearly demonstrating the survival benefits of RT for early-stage DLBCL. For OS, the HRs of RT vs. non-RT in the age groups of 18–40, 41–60, 61–80, and > 80 years were 0.43 (0.30–0.61), 0.68 (0.58–0.79), 0.67 (0.61–0.73), and 0.75 (0.66–0.85), respectively (Fig. 3a). For RS, the HRs of RT vs. non-RT were 0.45 (0.32–0.63), 0.68 (0.59–0.79), 0.68 (0.62–0.75), and 0.77 (0.67–0.87), respectively (Fig. 3b).

Considering the potential age-treatment interaction, RT vs. non-RT was associated with better OS (HR, 0.45; 95 % CI, 0.31–0.65; P < 0.001) (Fig. 3c) and RS (HR, 0.48; 95 % CI, 0.34–0.68; P < 0.001) (Fig. 3d), taking age as a continuous variable. Furthermore, a significant interaction between age and RT was identified in both OS (P_interaction_ = 0.020) and RS (P_interaction_ = 0.038) after adjusting through IPTW and multivariable analysis, suggesting greater benefit of RT in younger than in older patients.

### Linear association of OS and RS benefits of RT

**Figure 5:**
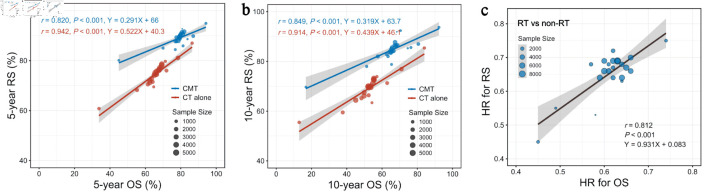
Linear association of OS and RS benefits at the treatment arm level. (a) Linear associations between 5-year OS and 5-year RS rates in subgroups stratified by clinical factors and age. (b) Linear associations between 10-year OS and 10-year RS rates in subgroups stratified by clinical factors and age. (c) Linear associations between HR (RT vs. non-RT) for OS and RS across subgroups stratified by clinical factors and age. CMT: combined-modality treatment; CT: chemotherapy; HR: hazard ratio; OS: overall survival; RS: relative survival; RT: radiotherapy.

Finally, we evaluated the correlation between OS and RS benefits of RT across subgroups stratified by clinical characteristics and age. At the treatment-arm level, 5-year OS rate correlated linearly with 5-year RS rate, regardless of CMT (r = 0.820, P < 0.001) (Fig. 5a) or chemotherapy alone (r = 0.942, P < 0.001). A similar linear correlation was observed between 10-year OS and RS rates according to the treatment administered, i.e., CMT (r = 0.849, P < 0.001) (Fig. 5b) or chemotherapy alone (r = 0.914, P < 0.001). The HR of RT vs. non-RT for OS correlated well with the RS benefit of RT across subgroups stratified by clinical characteristics and age (r = 0.812, P < 0.001) (Fig. 5c). This indicated that treatment gain of RT in RS could predict OS benefit with an acceptable consistency.

## Discussion

The heterogeneity of DLBCL and the high competing risk of non-LRDs in elderly patients make an assessment of the true survival benefit of RT challenging. In this population-based study, older age was found to be associated with poor prognosis when OS was used as the endpoint. However, similar results were not obtained for RS after controlling for background mortality. CMT resulted in higher long-term OS and RS rates and a lower SMR compared to chemotherapy alone across all ages. The interaction between age and RT indicated that younger patients derived greater survival benefits from RT than older patients. In addition, there was a linear correlation between RS and OS at the treatment arm level, indicating that RS is a valid alternative endpoint for prognostication and evaluation of treatment benefit. These findings suggested that additional RT improves net survival across all ages in early-stage DLBCL patients in the rituximab era, particularly in young adults.

Understanding the actual relationship between age and net survival benefit is important for prognostication and treatment selection in DLBCL. The patterns and incidence rates of LRDs and non-LRDs differed among the age groups. The risk of LRD and non-LRD increased with increasing age in early-stage DLBCL. However, patients aged ≤ 60 years consistently remained at a higher risk for mortality from DLBCL than from other causes after diagnosis, whereas those aged > 60 years were at a higher risk of mortality from other causes than from DLBCL beyond 10 years of follow-up. Although previous prognostic models have used old age at a cutoff point of 60 years as a risk factor in DLBCL [13, 14, 17, 19–21], we demonstrated a linear increase in mortality risk without an apparent age cutoff demarcating the OS difference. After controlling for background mortality, older age was no longer a poor prognostic factor for RS, indicating RS as a potentially superior endpoint to OS in the elderly population. Despite the biological heterogeneity between different patient ages [29–31], the assumption that elderly patients experience greater survival loss due to DLBCL compared to younger patients simply because of their advanced age is unjustified.

Previous studies have not specifically examined how age or background mortality affects the survival benefit of RT. In this study, CMT was associated with significantly better long-term survival rates than chemotherapy alone across all age groups, even after balancing for treatment selection bias and adjusting for covariates. The absolute gain in 10-year OS and RS rates for CMT vs. chemotherapy alone was approximately 10%. The most important finding of this study was that the RS rate of patients initially treated with CMT remained constantly high at approximately 95% at 10 years, with a plateau survival curve in young patients aged 18–40 years. Furthermore, the significant interaction between age and OS/RS suggested that additional RT provided significantly greater survival benefits in younger patients than in elderly patients. A possible explanation for this finding is that DLBCL patients are at constant risk for disease relapse after initial treatment [32–35], and younger patients, with longer life expectancies, experience more LRDs, making them more likely to benefit from local control of primary disease. Moreover, given the low competing risk of background mortality and the significant association of age with RT benefit, control of primary or bulky lesions may be particularly important in young patients. For elderly patients, first-line treatment options should be based on a comprehensive geriatric risk-benefit assessment [36]. Complete abandonment of RT during initial treatment in these patients may negatively influence the long-term net survival and should be approached with caution. Over the past two decades, radiation oncologists have attempted to optimize RT regimens through modern techniques, including involved-site intensify-modulated radiation therapy and low radiation doses (24–36 Gy) [37–39]. Mediastinal lymph nodes are rarely involved in DLBCL patients except in cases of primary mediastinal B-cell lymphoma. Consequently, involved-site RT does not increase the risk of cardiac mortality [40, 41]. Based on these findings, randomized controlled trials are needed to confirm the survival benefit of additional RT in early-stage patients with unfavorable prognoses [13, 42].

In predominantly elderly DLBCL patients, having a median age of > 60 years, background mortality rates vary with increasing age and follow-up time because of deaths related to causes other than lymphoma [43]. RS analysis controls for background mortality in an age-, sex-, and calendar year-matched general population, making it useful for assessing the net survival benefit of RT. This study further demonstrated a strong linear correlation between RS and OS at the treatment arm level. The HR of CMT vs. chemotherapy alone for RS correlated with that for OS across all subgroups, indicating that treatment gain of RT in RS could predict OS benefit with an acceptable consistency. Based on the results of this study, RS may be considered a valid alternative endpoint for prognostication and benefit evaluation, particularly in elderly patients.

Our study had several strengths. First, it was the first population-based study to assess the relationship between age and survival benefit of RT in early-stage DLBCL. The age-dependent RS benefit of RT for early-stage DLBCL was a unique finding that has not been reported previously. Even more importantly, the RS benefit of RT was greater in young patients, a demographic group with the highest concern for long-term RT-related deaths. Second, as an endpoint, RS was effectively used to adjust for background mortality, providing a more accurate assessment of treatment effectiveness and the true relationship between age and prognosis. Third, our data were derived from long-running cancer registries, making the results more generalizable than those from a single center. Our previous study demonstrated that long-term survivorship surveillance is necessary to adequately assess treatment benefits for early-stage DLBCL [3].

There were several limitations of this study, mainly related to data availability from the SEER database. First, there was a lack of important clinical variables related to the selection or avoidance of RT, such as performance status, extranodal and bulky diseases, International Prognostic Index scores, serum lactate dehydrogenase levels, and comorbidities. Second, the absence of detailed information on immunochemotherapy prevented us from determining which patients received R-CHOP. Third, there were difficulties in controlling for residual confounders while evaluating the treatment efficacy in a retrospective study. Therefore, the findings of this study need to be validated through well-designed randomized trials.

In conclusion, initial CMT resulted in better OS and RS, along with a lower SMR, compared to chemotherapy alone across all age groups. RS proved to be a valid endpoint, equivalent to OS, in early-stage DLBCL. These findings highlight the potential role of consolidative RT as a part of first-line treatment for early-stage DLBCL in the rituximab era, particularly in young adults.

## Supplementary Material

Suppl 1 Natural spline plot for SMR (CMT vs. chemotherapy alone) according to age at diagnosis.

Suppl 2 Subgroup analyses for OS and RS. The forest plot depicts HRs for OS (A) and RS (B) among patients treated with CMT vs. chemotherapy alone.

## Data Availability

The data supporting this study’s findings are available in the public domain (http://seer.cancer.gov/).

## Abbreviations

CI: confidence interval
CMT: combined-modality treatment
DLBCL: diffuse large B-cell lymphoma
HR: hazard ratio
IPI: International Prognostic Index
IPTW: inverse probability of treatment weighting
LRD: lymphoma-related death
NHL: non-Hodgkin lymphoma
OS: overall survival
PFS: progression-free survival
PS: propensity scores
RS: relative survival
RT: radiotherapy
SEER: Surveillance, Epidemiology, and End Results
SMR: standardized mortality ratio

## References

[1] Sehn LH, Salles G (2021). Diffuse large B-cell lymphoma. N Engl J Med.

[2] Hawkes EA, Barraclough A, Sehn LH (2022). Limited-stage diffuse large B-cell lymphoma. Blood.

[3] Wang X, Liu X, Zhong QZ, Wu T, Wu YP, Yang Y, Chen B (2023). Decreased lymphoma-related deaths and improved long-term relative survival with radiotherapy for early-stage diffuse large B-cell lymphoma in the rituximab era. Radiother Oncol.

[4] https://www.nccn.org/professionals/physician_gls/pdf/b-cell.pdf.

[5] Fox CP, Chaganti S, McIlroy G, Barrington SF, Burton C, Cwynarski K, Eyre TA (2024). The management of newly diagnosed large B-cell lymphoma: A British Society for Haematology Guideline. Br J Haematol.

[6] Tilly H, Gomes da Silva M, Vitolo U, Jack A, Meignan M, Lopez-Guillermo A, Walewski J (2015). Diffuse large B-cell lymphoma (DLBCL): ESMO Clinical Practice Guidelines for diagnosis, treatment and follow-up. Ann Oncol.

[7] Guma J, Palazon-Carrion N, Rueda-Dominguez A, Sequero S, Calvo V, Garcia-Arroyo R, Gomez-Codina J (2025). SEOM-GOTEL clinical guidelines on diffuse large B-cell lymphoma (update 2025). Clin Transl Oncol.

[8] Zhuang D, Huang S, Zhang P, Lei D, Wang Y, Liu H, Nie M (2025). Efficacy of radiotherapy in primary mediastinal diffuse large B-cell lymphoma: a systematic review and meta-analysis of 1,392 patients. Ann Hematol.

[9] Lamy T, Damaj G, Soubeyran P, Gyan E, Cartron G, Bouabdallah K, Gressin R (2018). R-CHOP 14 with or without radiotherapy in nonbulky limited-stage diffuse large B-cell lymphoma. Blood.

[10] Persky DO, Li H, Stephens DM, Park SI, Bartlett NL, Swinnen LJ, Barr PM (2020). Positron emission tomography-directed therapy for patients with limited-stage diffuse large B-cell lymphoma: results of intergroup national clinical trials network study S1001. J Clin Oncol.

[11] Poeschel V, Held G, Ziepert M, Witzens-Harig M, Holte H, Thurner L, Borchmann P (2019). Four versus six cycles of CHOP chemotherapy in combination with six applications of rituximab in patients with aggressive B-cell lymphoma with favourable prognosis (FLYER): a randomised, phase 3, non-inferiority trial. Lancet.

[12] Dabaja BS, Vanderplas AM, Crosby-Thompson AL, Abel GA, Czuczman MS, Friedberg JW, Gordon LI (2015). Radiation for diffuse large B-cell lymphoma in the rituximab era: analysis of the National Comprehensive Cancer Network lymphoma outcomes project. Cancer.

[13] Haque W, Dabaja B, Tann A, Khan M, Szeja S, Butler EB, Teh BS (2016). Changes in treatment patterns and impact of radiotherapy for early stage diffuse large B cell lymphoma after Rituximab: A population-based analysis. Radiother Oncol.

[14] Vargo JA, Gill BS, Balasubramani GK, Beriwal S (2015). Treatment selection and survival outcomes in early-stage diffuse large B-cell lymphoma: Do we still need consolidative radiotherapy?. J Clin Oncol.

[15] Wang J, Liu X, Wu Y, Zhong Q, Wu T, Yang Y, Chen B (2024). Association of overall survival benefit of radiotherapy with progression-free survival after chemotherapy for diffuse large B-cell lymphoma: A systematic review and meta-analysis. J Natl Cancer Cent.

[16] Abu Sabaa A, Morth C, Hasselblom S, Hedstrom G, Flogegard M, Stern M, Andersson PO (2021). Age is the most important predictor of survival in diffuse large B-cell lymphoma patients achieving event-free survival at 24 months: a Swedish population-based study. Br J Haematol.

[17] Mikhaeel NG, Heymans MW, Eertink JJ, de Vet HCW, Boellaard R, Duhrsen U, Ceriani L (2022). Proposed new dynamic prognostic index for diffuse large B-cell lymphoma: international metabolic prognostic index. J Clin Oncol.

[18] Pal SK, Hurria A (2010). Impact of age, sex, and comorbidity on cancer therapy and disease progression. J Clin Oncol.

[19] International Non-Hodgkin’s Lymphoma Prognostic Factors Project (1993). A predictive model for aggressive non-Hodgkin's lymphoma. N Engl J Med.

[20] Sehn LH, Berry B, Chhanabhai M, Fitzgerald C, Gill K, Hoskins P, Klasa R (2007). The revised International Prognostic Index (R-IPI) is a better predictor of outcome than the standard IPI for patients with diffuse large B-cell lymphoma treated with R-CHOP. Blood.

[21] Zhou Z, Sehn LH, Rademaker AW, Gordon LI, Lacasce AS, Crosby-Thompson A, Vanderplas A (2014). An enhanced International Prognostic Index (NCCN-IPI) for patients with diffuse large B-cell lymphoma treated in the rituximab era. Blood.

[22] Bright CJ, Brentnall AR, Wooldrage K, Myles J, Sasieni P, Duffy SW (2020). Errors in determination of net survival: cause-specific and relative survival settings. Br J Cancer.

[23] Perme MP, Stare J, Esteve J (2012). On estimation in relative survival. Biometrics.

[24] Ederer F, Axtell LM, Cutler SJ (1961). The relative survival rate: a statistical methodology. Natl Cancer Inst Monogr.

[25] McCaffrey DF, Griffin BA, Almirall D, Slaughter ME, Ramchand R, Burgette LF (2013). A tutorial on propensity score estimation for multiple treatments using generalized boosted models. Stat Med.

[26] Andrade C (2020). Mean difference, Standardized Mean Difference (SMD), and their use in meta-analysis: As simple as it gets. J Clin Psychiatry.

[27] Stare J, Henderson R, Pohar M (2005). An individual measure of relative survival. J Royal Stat Soc: Ser C (Appl Stats).

[28] Brambor T, Clark WR, Golder M (2006). Understanding interaction models: improving empirical analyses. Polit Anal.

[29] Thunberg U, Amini RM, Linderoth J, Roos G, Enblad G, Berglund M (2009). BCL2 expression in de novo diffuse large B-cell lymphoma partly reflects normal differences in age distribution. Br J Haematol.

[30] Mareschal S, Lanic H, Ruminy P, Bastard C, Tilly H, Jardin F (2011). The proportion of activated B-cell like subtype among de novo diffuse large B-cell lymphoma increases with age. Haematologica.

[31] Klapper W, Kreuz M, Kohler CW, Burkhardt B, Szczepanowski M, Salaverria I, Hummel M (2012). Patient age at diagnosis is associated with the molecular characteristics of diffuse large B-cell lymphoma. Blood.

[32] Bobillo S, Joffe E, Lavery JA, Sermer D, Ghione P, Noy A, Caron PC (2021). Clinical characteristics and outcomes of extranodal stage I diffuse large B-cell lymphoma in the rituximab era. Blood.

[33] Howlader N, Mariotto AB, Besson C, Suneja G, Robien K, Younes N, Engels EA (2017). Cancer-specific mortality, cure fraction, and noncancer causes of death among diffuse large B-cell lymphoma patients in the immunochemotherapy era. Cancer.

[34] Lugtenburg PJ, de Nully Brown P, van der Holt B, D'Amore FA, Koene HR, de Jongh E, Fijnheer R (2020). Rituximab-CHOP with early rituximab intensification for diffuse large B-cell lymphoma: a randomized phase III trial of the HOVON and the Nordic Lymphoma Group (HOVON-84). J Clin Oncol.

[35] Stephens DM, Li H, LeBlanc ML, Puvvada SD, Persky D, Friedberg JW, Smith SM (2016). Continued risk of relapse independent of treatment modality in limited-stage diffuse large B-cell lymphoma: final and long-term analysis of southwest oncology group study S8736. J Clin Oncol.

[36] Merli F, Luminari S, Tucci A, Arcari A, Rigacci L, Hawkes E, Chiattone CS (2021). Simplified geriatric assessment in older patients with diffuse large B-cell lymphoma: the prospective elderly project of the fondazione italiana linfomi. J Clin Oncol.

[37] Illidge T, Specht L, Yahalom J, Aleman B, Berthelsen AK, Constine L, Dabaja B (2014). Modern radiation therapy for nodal non-Hodgkin lymphoma-target definition and dose guidelines from the International Lymphoma Radiation Oncology Group. Int J Radiat Oncol Biol Phys.

[38] Lowry L, Smith P, Qian W, Falk S, Benstead K, Illidge T, Linch D (2011). Reduced dose radiotherapy for local control in non-Hodgkin lymphoma: a randomised phase III trial. Radiother Oncol.

[39] Wirth A, Mikhaeel NG, Aleman BMP, Pinnix CC, Constine LS, Ricardi U, Illidge TM (2020). Involved site radiation therapy in adult lymphomas: an overview of international lymphoma radiation oncology group guidelines. Int J Radiat Oncol Biol Phys.

[40] Parsons MW, Rock C, Chipman JJ, Shah HR, Hu B, Stephens DM, Tao R (2023). Secondary malignancies in non-Hodgkin lymphoma survivors: 40 years of follow-up assessed by treatment modality. Cancer Med.

[41] Pugh TJ, Ballonoff A, Rusthoven KE, McCammon R, Kavanagh B, Newman F, Rabinovitch R (2010). Cardiac mortality in patients with stage I and II diffuse large B-cell lymphoma treated with and without radiation: a surveillance, epidemiology, and end-results analysis. Int J Radiat Oncol Biol Phys.

[42] Oertel M, Ziepert M, Frontzek F, Nacke N, Altmann B, Nickelsen M, Glass B (2024). Radiotherapy in younger patients with advanced aggressive B-cell lymphoma-long-term results from the phase 3 R-MegaCHOEP trial. Leukemia.

[43] Gong IY, Crump M, Prica A, Calzavara A, Liu N, Kordbacheh T, Rodin D (2025). Outcomes and factors influencing survival in patients with diffuse large B-cell lymphoma: a population-based analysis. Blood Neoplasia.

