# Supplementary material for: Interactive Effect of Age on Overall and Relative Survival Benefits of Radiotherapy for Early-Stage Diffuse Large B-Cell Lymphoma in the Rituximab Era
Source: J Hematol. 2026 Feb 20;15(1):34–44. doi: 10.14740/jh2134 (PMC12948474; doi:10.14740/jh2134)

**Suppl 1.** Natural spline plot for SMR (CMT vs. chemotherapy alone) according to age at diagnosis. CMT, combined-modality treatment; SMR, standardized mortality ratio.


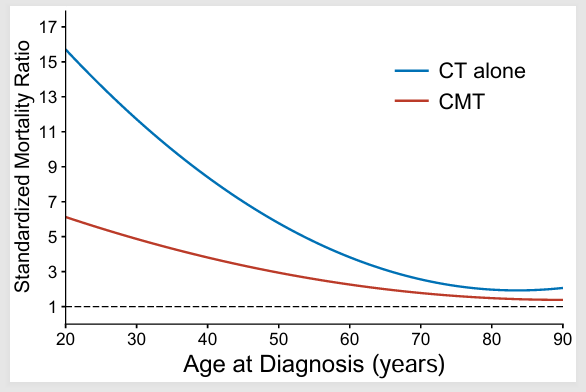

Supplement: Suppl 1 — Natural spline plot for SMR (CMT vs. chemotherapy alone) according to age at diagnosis. [file jh-15-01-034-s001.docx]
