# Supplementary material for: Interactive Effect of Age on Overall and Relative Survival Benefits of Radiotherapy for Early-Stage Diffuse Large B-Cell Lymphoma in the Rituximab Era
Source: J Hematol. 2026 Feb 20;15(1):34–44. doi: 10.14740/jh2134 (PMC12948474; doi:10.14740/jh2134)

**Suppl 2.** Subgroup analyses for OS and RS. The Forest plot depicts HRs for OS (A) and RS (B) among patients treated with CMT vs. chemotherapy alone. OS, overall survival; RS, relative survival; HR, hazard ratio; CMT, combined-modality treatment.


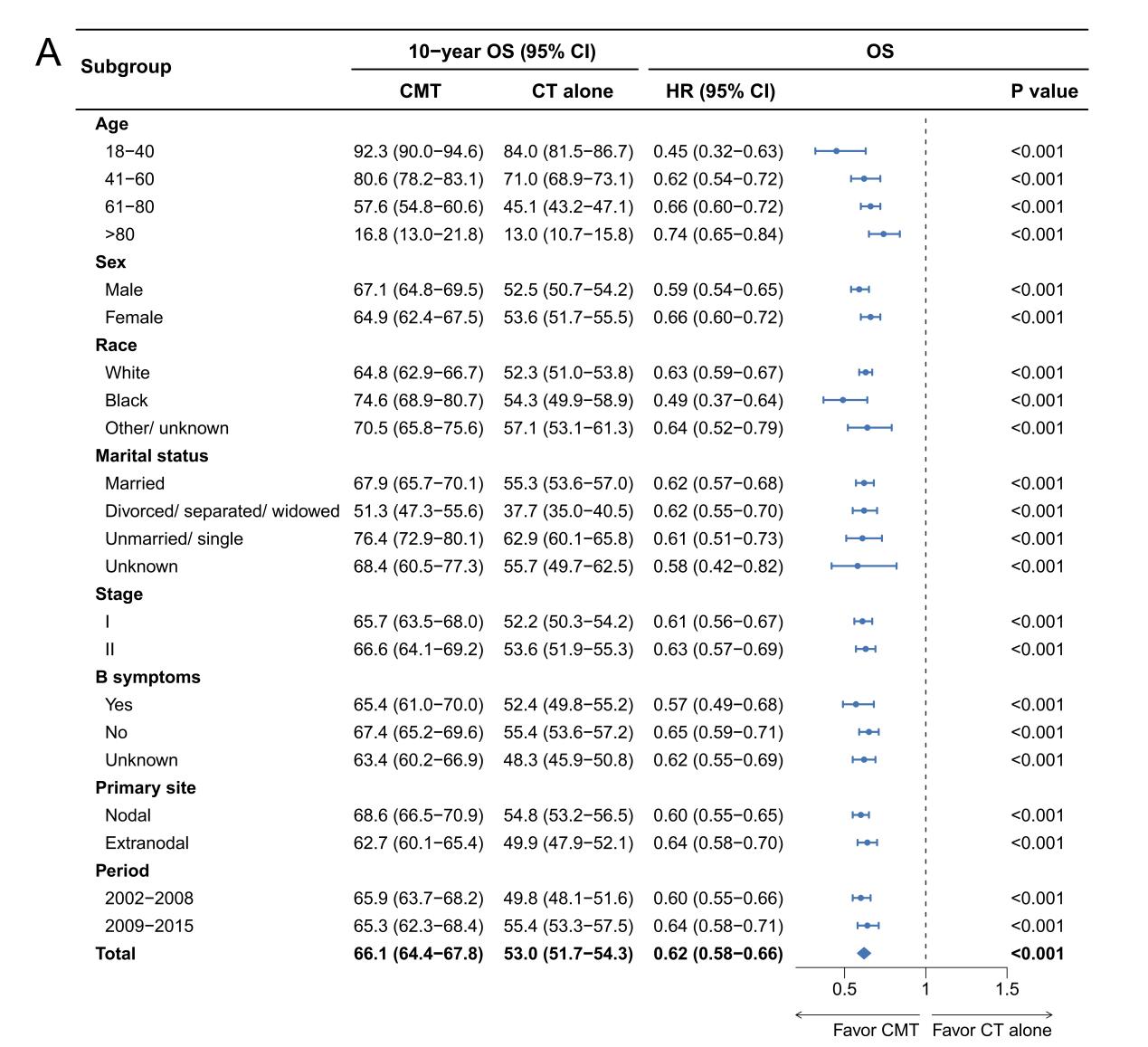


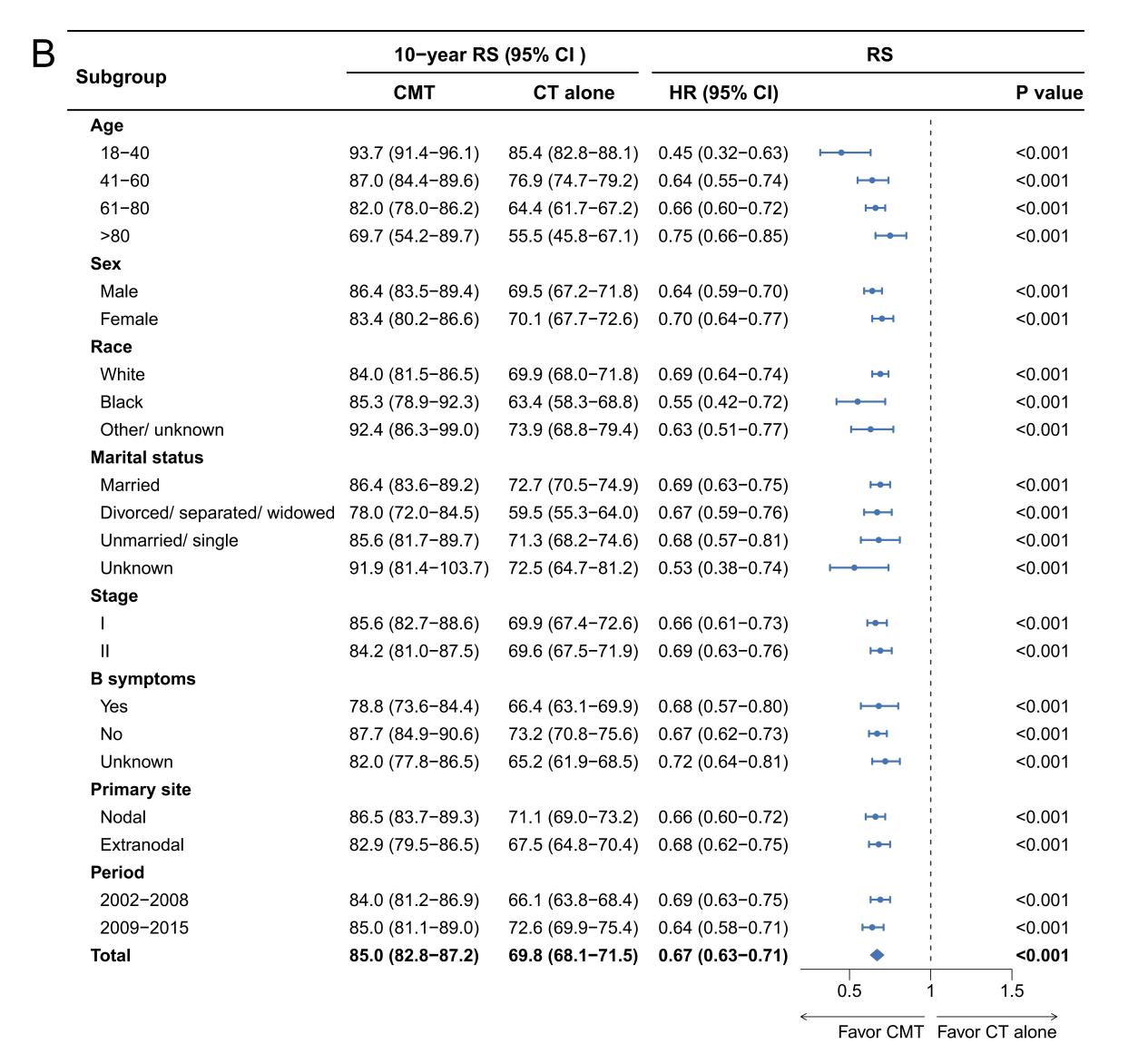

Supplement: Suppl 2 — Subgroup analyses for OS and RS. The forest plot depicts HRs for OS (A) and RS (B) among patients treated with CMT vs. chemotherapy alone. [file jh-15-01-034-s002.docx]
